# 4-({[(*E*)-Pyridin-3-yl­methyl­idene]amino}­meth­yl)cyclo­hexa­necarb­oxy­lic acid

**DOI:** 10.1107/S1600536811011779

**Published:** 2011-04-07

**Authors:** Muhammad Nisar, Ihsan Ali, M. Nawaz Tahir, Mughal Qayum, Inamullah Khan Marwat

**Affiliations:** a Institute of Chemical Sciences, University of Peshawar, Peshawar 25120, Pakistan; b Department of Physics, University of Sargodha, Sargodha, Pakistan; c Department of Pharmacy, University of Peshawar, Peshawar 25120, Pakistan

## Abstract

The title compound, C_14_H_18_N_2_O_2_, contains two geometrically different mol­ecules in the asymmetric unit: the basal plane of the cyclo­hexane chair and the *N*-[pyridin-3-yl­methyl­idene]methanamine moiety are oriented at dihedral angles of 71.77 (7)° and 83.42 (8)°. In the crystal, the mol­ecules are linked by O—H⋯N hydrogen bonds, generating *C*(13) head-to-tail chains extending along the base vector [103]. *R*_2_^2^(26) ring motifs are formed due to the C—H⋯·O inter­actions that link neighbouring chains. There also exist π–π inter­actions [centroid–centroid separation = 3.6925 (12) Å] between the symmetry-related pyridine rings of one of the independent mol­ecules.

## Related literature

For related structures, see: Huh & Lee (2007 ▶): Shahzadi *et al.* (2007 ▶). For graph-set notation, see: Bernstein *et al.* (1995 ▶).
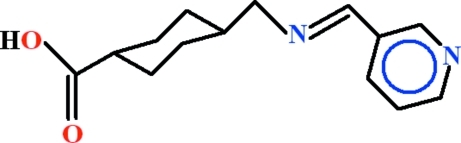


## Experimental

### 

#### Crystal data

C_14_H_18_N_2_O_2_
*M* *_r_* = 246.31
Monoclinic, 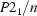

*a* = 12.7580 (6) Å
*b* = 11.2504 (6) Å
*c* = 18.8088 (7) Å
β = 94.720 (2)°
*V* = 2690.5 (2) Å^3^
*Z* = 8
Mo *K*α radiation
μ = 0.08 mm^−1^
*T* = 296 K
0.34 × 0.25 × 0.22 mm

#### Data collection

Bruker Kappa APEXII CCD diffractometer
Absorption correction: multi-scan (*SADABS*; Bruker, 2005 ▶) *T*_min_ = 0.975, *T*_max_ = 0.983
24311 measured reflections
6635 independent reflections
3908 reflections with *I* > 2σ(*I*)
*R*_int_ = 0.034

#### Refinement

*R*[*F* ^2^ > 2σ(*F* ^2^)] = 0.063
*wR*(*F* ^2^) = 0.207
*S* = 1.05
6635 reflections
327 parameters
H-atom parameters constrained
Δρ_max_ = 0.50 e Å^−3^
Δρ_min_ = −0.25 e Å^−3^

### 

Data collection: *APEX2* (Bruker, 2009 ▶); cell refinement: *SAINT* (Bruker, 2009 ▶); data reduction: *SAINT*; program(s) used to solve structure: *SHELXS97* (Sheldrick, 2008 ▶); program(s) used to refine structure: *SHELXL97* (Sheldrick, 2008 ▶); molecular graphics: *ORTEP-3 for Windows* (Farrugia, 1997 ▶) and *PLATON* (Spek, 2009 ▶); software used to prepare material for publication: *WinGX* (Farrugia, 1999 ▶) and *PLATON*.

## Supplementary Material

Crystal structure: contains datablocks global, I. DOI: 10.1107/S1600536811011779/hb5830sup1.cif

Structure factors: contains datablocks I. DOI: 10.1107/S1600536811011779/hb5830Isup2.hkl

Additional supplementary materials:  crystallographic information; 3D view; checkCIF report

## Figures and Tables

**Table 1:** Hydrogen-bond geometry (Å, °)

*D*—H⋯*A*	*D*—H	H⋯*A*	*D*⋯*A*	*D*—H⋯*A*
O1—H1⋯N4^i^	0.82	1.87	2.682 (2)	171
O3—H3⋯N2^ii^	0.82	1.89	2.685 (2)	164
C11—H11⋯O2^iii^	0.93	2.56	3.478 (3)	168
C13—H13⋯O2^iv^	0.93	2.57	3.316 (3)	137
C27—H27⋯O4^v^	0.93	2.45	3.280 (3)	148

Symmetry codes: (i) 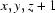; (ii) 
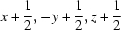
; (iii) 
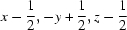
; (iv) 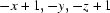; (v) 
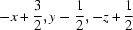
.

**Fig. 1.**
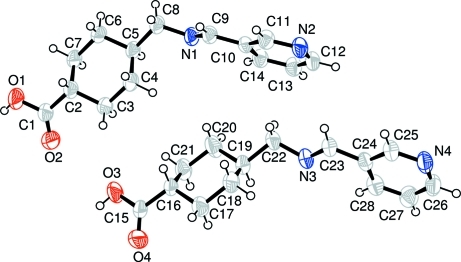
View of the title compound with displacement ellipsoids drawn at the 50% probability level. H-atoms are shown as small spheres of arbitrary radius.

**Fig. 2.**
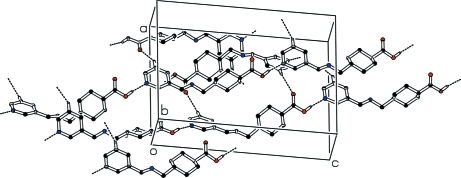
The partial packing, which shows that the molecules form polymeric chains and ring motifs.

## supplementary crystallographic information

### Comment

The title compound (I, Fig. 1) has been prepared for the study of biological studies and for the synthesis of metallic complexes.

The crystal structure of (II) *i.e.*, 4-(aminomethyl)cyclohexane-1-carboxylic acid (Shahzadi *et al.*, 2007) and (III) *i.e.*, *N*,*N*^'^-bis(pyridin-3-ylmethylene)cyclohexane-*trans*-1,4-diamine (Huh & Lee, 2007) have been published which are related to the title compound.

The title compound consists of two molecules in the crystallographic asymmetric unit which differ from each other geometrically. In one molecules, the basal plane A (C3/C4/C6/C7) of cyclohexane and *N*-[pyridin-3-ylmethylidene]methanamine moiety B (C8—C14/N1/N2) are almost planar with r.m.s. deviation of 0.014 and 0.034 Å, respectively. The dihedral angle between A/B is 71.77 (7)°. The carboxylate group C (O1/C1/O2) is of course planar. The dihedral angle between A/C and B/C is 30.07 (15)° and 53.36 (15)°, respectively. In second molecules, the basal plane D (C17/C18/C20/C21) of cyclohexane and *N*-[pyridin-3-ylmethylidene]methanamine moiety E (C22—C28/N3/N4) are also almost planar with r.m.s. deviation of 0.006 and 0.047 Å, respectively. The dihedral angle between D/E is 83.42 (8)°. The carboxylate group F (O3/C15/O4) makes dihedral angle of 30.03 (26)° and 62.40 (14)° with D and E, respectively.

In the crystal, the molecules are stabilized in the form of infinite C(13) polymeric chains due to O—H···.N H-bonds (Table 1, Fig. 2) extending along the base vector [103]. Due to intermolecular H-bonding of C—H···.O type (Table 1, Fig. 2) ring motifs (Bernstein *et al.*, 1995) *R*_2_^2^(26) are formed. The molecules are further stabilized by the π···π interaction between the symmetry related pyridine ring (C24/C25/N4/C26/C27/C28) at a distance of 3.6925 (12) Å.

### Experimental

A two-necked reaction flask equipped with a reflux condenser, serum cap and a magnet bar was charged with a methanolic solution (40 ml) of tranexamic acid (0.157 g, 1 mmol) and pyridine-3-carboxaldehyde (0.107 g, 1 mmol) at room temperature under nitrogen atmosphere. An excess amount of triethylamine (1 ml) was dropped into the reaction mixture through a serum cap and subsequently the reaction mixture was refluxed for about 20 h. The disappearance of the starting materials was ascertained by TLC (methanol:chloroform). After completion of the reaction, an equivalent quantity of glacial acetic acid was added to the mixture to ensure neutralization of triethylamine. Later on, the crude mixture was allowed to stand overnight which resulted gradually into crystallized material. The solid was collected by suction filtration, washed with diethyl ether and recrystalized from hot methanol to give colourless prisms of (I).

### Refinement

The coordinates of H-atoms of hydroxy groups were refined. The H-atoms were positioned geometrically (O—H = 0.82, C–H = 0.93—0.98 Å) and refined as riding with *U*_iso_(H) = x*U*_eq_(C, O), where x = 1.2 for all H-atoms.

### Crystal data

**Table:**

C_14_H_18_N_2_O_2_	*F*(000) = 1056
*M**_r_* = 246.31	*D*_x_ = 1.216 Mg m^−^^3^
Monoclinic, *P*2_1_/*n*	Mo *K*α radiation, λ = 0.71073 Å
Hall symbol: -P 2yn	Cell parameters from 3908 reflections
*a* = 12.7580 (6) Å	θ = 1.9–28.3°
*b* = 11.2504 (6) Å	µ = 0.08 mm^−^^1^
*c* = 18.8088 (7) Å	*T* = 296 K
β = 94.720 (2)°	Prism, colourless
*V* = 2690.5 (2) Å^3^	0.34 × 0.25 × 0.22 mm
*Z* = 8	

### Data collection

**Table:**

Bruker Kappa APEXII CCD diffractometer	6635 independent reflections
Radiation source: fine-focus sealed tube	3908 reflections with *I* > 2σ(*I*)
graphite	*R*_int_ = 0.034
Detector resolution: 7.50 pixels mm^-1^	θ_max_ = 28.3°, θ_min_ = 1.9°
ω scans	*h* = −15→17
Absorption correction: multi-scan (*SADABS*; Bruker, 2005)	*k* = −13→14
*T*_min_ = 0.975, *T*_max_ = 0.983	*l* = −25→21
24311 measured reflections	

### Refinement

**Table:**

Refinement on *F*^2^	Primary atom site location: structure-invariant direct methods
Least-squares matrix: full	Secondary atom site location: difference Fourier map
*R*[*F*^2^ > 2σ(*F*^2^)] = 0.063	Hydrogen site location: inferred from neighbouring sites
*wR*(*F*^2^) = 0.207	H-atom parameters constrained
*S* = 1.05	*w* = 1/[σ^2^(*F*_o_^2^) + (0.1099*P*)^2^ + 0.2692*P*] where *P* = (*F*_o_^2^ + 2*F*_c_^2^)/3
6635 reflections	(Δ/σ)_max_ < 0.001
327 parameters	Δρ_max_ = 0.50 e Å^−^^3^
0 restraints	Δρ_min_ = −0.25 e Å^−^^3^

### Special details

**Table:**

Geometry. Bond distances, angles *etc*. have been calculated using the rounded fractional coordinates. All su's are estimated from the variances of the (full) variance-covariance matrix. The cell e.s.d.'s are taken into account in the estimation of distances, angles and torsion angles
Refinement. Refinement of *F*^2^ against ALL reflections. The weighted *R*-factor *wR* and goodness of fit *S* are based on *F*^2^, conventional *R*-factors *R* are based on *F*, with *F* set to zero for negative *F*^2^. The threshold expression of *F*^2^ > σ(*F*^2^) is used only for calculating *R*-factors(gt) *etc*. and is not relevant to the choice of reflections for refinement. *R*-factors based on *F*^2^ are statistically about twice as large as those based on *F*, and *R*- factors based on ALL data will be even larger.

### Fractional atomic coordinates and isotropic or equivalent isotropic displacement parameters (Å^2^)

**Table:**

	*x*	*y*	*z*	*U*_iso_*/*U*_eq_	
O1	0.34542 (12)	0.20319 (19)	0.85240 (7)	0.0774 (7)	
O2	0.48692 (13)	0.19021 (19)	0.79343 (8)	0.0852 (8)	
N1	0.18226 (12)	−0.02615 (17)	0.48003 (7)	0.0500 (6)	
N2	0.18135 (14)	0.04146 (17)	0.22905 (8)	0.0552 (6)	
C1	0.39348 (17)	0.1868 (2)	0.79392 (10)	0.0542 (7)	
C2	0.31681 (15)	0.1641 (2)	0.72955 (9)	0.0496 (7)	
C3	0.37171 (17)	0.1506 (2)	0.66075 (9)	0.0607 (8)	
C4	0.29250 (17)	0.1273 (2)	0.59682 (9)	0.0577 (8)	
C5	0.22042 (14)	0.0229 (2)	0.60795 (8)	0.0465 (6)	
C6	0.16868 (15)	0.0366 (2)	0.67770 (9)	0.0558 (7)	
C7	0.24956 (15)	0.0556 (2)	0.74099 (9)	0.0529 (7)	
C8	0.13716 (15)	0.0072 (2)	0.54600 (9)	0.0543 (7)	
C9	0.15528 (15)	0.0323 (2)	0.42457 (9)	0.0482 (6)	
C10	0.19220 (14)	0.00271 (18)	0.35472 (8)	0.0429 (6)	
C11	0.15284 (15)	0.0644 (2)	0.29463 (9)	0.0509 (7)	
C12	0.25168 (16)	−0.0442 (2)	0.22215 (10)	0.0552 (7)	
C13	0.29540 (16)	−0.1099 (2)	0.27881 (10)	0.0547 (7)	
C14	0.26511 (15)	−0.08693 (19)	0.34589 (9)	0.0493 (6)	
O3	0.58371 (14)	0.35232 (17)	0.61410 (7)	0.0740 (7)	
O4	0.69327 (16)	0.4490 (2)	0.55311 (8)	0.1074 (8)	
N3	0.50418 (13)	0.15359 (17)	0.22618 (8)	0.0547 (6)	
N4	0.47454 (15)	0.19104 (17)	−0.02788 (8)	0.0584 (6)	
C15	0.61952 (15)	0.3860 (2)	0.55416 (9)	0.0493 (7)	
C16	0.55551 (15)	0.33872 (19)	0.48915 (9)	0.0470 (6)	
C17	0.59449 (19)	0.3837 (2)	0.41964 (10)	0.0636 (8)	
C18	0.52518 (18)	0.3364 (2)	0.35534 (10)	0.0600 (8)	
C19	0.52023 (17)	0.2027 (2)	0.35542 (9)	0.0535 (7)	
C20	0.4829 (2)	0.1581 (3)	0.42478 (11)	0.0726 (9)	
C21	0.55003 (19)	0.2050 (2)	0.48971 (10)	0.0622 (8)	
C22	0.45076 (17)	0.1549 (2)	0.29154 (9)	0.0612 (8)	
C23	0.45152 (16)	0.17579 (19)	0.16841 (9)	0.0485 (6)	
C24	0.49662 (15)	0.16380 (18)	0.09908 (9)	0.0460 (6)	
C25	0.44008 (17)	0.2013 (2)	0.03678 (9)	0.0522 (7)	
C26	0.56779 (19)	0.1403 (2)	−0.03228 (11)	0.0623 (8)	
C27	0.62979 (18)	0.0993 (2)	0.02655 (12)	0.0650 (8)	
C28	0.59361 (16)	0.1115 (2)	0.09252 (10)	0.0564 (7)	
H1	0.38897	0.20427	0.88698	0.0929*	
H2	0.26983	0.23300	0.72381	0.0596*	
H3A	0.41080	0.22255	0.65240	0.0729*	
H3B	0.42131	0.08524	0.66588	0.0729*	
H4A	0.25009	0.19799	0.58746	0.0692*	
H4B	0.33044	0.11217	0.55512	0.0692*	
H5	0.26354	−0.04928	0.61141	0.0557*	
H6A	0.12781	−0.03406	0.68586	0.0670*	
H6B	0.12088	0.10380	0.67389	0.0670*	
H7A	0.21368	0.06593	0.78407	0.0634*	
H7B	0.29423	−0.01402	0.74715	0.0634*	
H8A	0.08785	−0.05372	0.55814	0.0652*	
H8B	0.09846	0.08095	0.53848	0.0652*	
H9	0.11027	0.09675	0.42786	0.0579*	
H11	0.10427	0.12470	0.30007	0.0611*	
H12	0.27229	−0.06053	0.17687	0.0663*	
H13	0.34461	−0.16888	0.27181	0.0657*	
H14	0.29300	−0.13074	0.38490	0.0591*	
H3	0.62309	0.37640	0.64768	0.0888*	
H16	0.48356	0.36827	0.49122	0.0563*	
H17A	0.66650	0.35801	0.41633	0.0763*	
H17B	0.59343	0.46992	0.41923	0.0763*	
H18A	0.45469	0.36834	0.35640	0.0720*	
H18B	0.55315	0.36332	0.31170	0.0720*	
H19	0.59168	0.17263	0.35182	0.0642*	
H20A	0.48481	0.07191	0.42511	0.0871*	
H20B	0.41048	0.18253	0.42792	0.0871*	
H21A	0.52030	0.17872	0.53287	0.0747*	
H21B	0.62051	0.17260	0.48987	0.0747*	
H22A	0.42867	0.07476	0.30202	0.0734*	
H22B	0.38817	0.20383	0.28439	0.0734*	
H23	0.38208	0.20049	0.16936	0.0582*	
H25	0.37442	0.23562	0.04046	0.0626*	
H26	0.59272	0.13195	−0.07710	0.0748*	
H27	0.69467	0.06412	0.02123	0.0779*	
H28	0.63391	0.08479	0.13282	0.0676*	

### Atomic displacement parameters (Å^2^)

**Table:**

	*U*^11^	*U*^22^	*U*^33^	*U*^12^	*U*^13^	*U*^23^
O1	0.0682 (10)	0.1258 (17)	0.0379 (7)	0.0013 (10)	0.0026 (7)	−0.0168 (9)
O2	0.0607 (10)	0.1432 (19)	0.0519 (9)	−0.0195 (10)	0.0063 (7)	−0.0162 (10)
N1	0.0513 (9)	0.0658 (12)	0.0323 (7)	0.0041 (8)	−0.0004 (6)	−0.0019 (7)
N2	0.0652 (10)	0.0674 (13)	0.0318 (7)	−0.0006 (9)	−0.0030 (7)	0.0051 (8)
C1	0.0618 (13)	0.0623 (15)	0.0390 (10)	−0.0043 (10)	0.0075 (9)	−0.0034 (9)
C2	0.0545 (11)	0.0606 (14)	0.0339 (9)	0.0018 (9)	0.0042 (8)	−0.0028 (9)
C3	0.0612 (12)	0.0862 (18)	0.0357 (10)	−0.0198 (11)	0.0095 (9)	−0.0031 (10)
C4	0.0648 (12)	0.0790 (17)	0.0300 (9)	−0.0088 (11)	0.0088 (8)	0.0053 (9)
C5	0.0451 (10)	0.0635 (14)	0.0307 (8)	0.0066 (8)	0.0026 (7)	0.0014 (8)
C6	0.0479 (10)	0.0848 (17)	0.0354 (9)	−0.0032 (10)	0.0077 (8)	0.0001 (10)
C7	0.0559 (11)	0.0744 (16)	0.0288 (8)	−0.0033 (10)	0.0064 (8)	0.0036 (9)
C8	0.0497 (10)	0.0801 (16)	0.0331 (9)	0.0061 (10)	0.0035 (8)	−0.0016 (9)
C9	0.0478 (10)	0.0583 (13)	0.0380 (9)	0.0040 (9)	0.0005 (7)	−0.0010 (9)
C10	0.0437 (9)	0.0510 (12)	0.0332 (8)	−0.0027 (8)	−0.0016 (7)	0.0015 (8)
C11	0.0539 (11)	0.0581 (14)	0.0400 (9)	0.0072 (9)	−0.0009 (8)	0.0065 (9)
C12	0.0651 (13)	0.0657 (15)	0.0349 (9)	−0.0087 (10)	0.0041 (9)	−0.0056 (9)
C13	0.0585 (12)	0.0562 (14)	0.0495 (11)	0.0053 (10)	0.0044 (9)	−0.0064 (10)
C14	0.0544 (11)	0.0548 (13)	0.0379 (9)	0.0038 (9)	−0.0010 (8)	0.0050 (9)
O3	0.0978 (12)	0.0927 (14)	0.0312 (7)	−0.0347 (10)	0.0032 (7)	−0.0069 (8)
O4	0.0983 (13)	0.179 (2)	0.0455 (9)	−0.0758 (15)	0.0102 (8)	−0.0169 (11)
N3	0.0624 (10)	0.0665 (12)	0.0341 (8)	−0.0035 (8)	−0.0021 (7)	−0.0072 (8)
N4	0.0747 (12)	0.0651 (13)	0.0346 (8)	−0.0048 (9)	−0.0001 (8)	−0.0044 (8)
C15	0.0515 (11)	0.0640 (14)	0.0327 (9)	−0.0015 (9)	0.0060 (8)	−0.0063 (9)
C16	0.0475 (10)	0.0619 (14)	0.0316 (8)	−0.0011 (9)	0.0040 (7)	−0.0061 (8)
C17	0.0808 (15)	0.0708 (16)	0.0391 (10)	−0.0209 (12)	0.0044 (10)	−0.0015 (10)
C18	0.0707 (13)	0.0744 (17)	0.0346 (9)	−0.0040 (11)	0.0020 (9)	0.0045 (10)
C19	0.0595 (12)	0.0672 (15)	0.0336 (9)	−0.0026 (10)	0.0020 (8)	−0.0062 (9)
C20	0.1047 (19)	0.0713 (17)	0.0401 (10)	−0.0256 (14)	−0.0037 (11)	0.0018 (10)
C21	0.0824 (15)	0.0681 (16)	0.0353 (9)	−0.0030 (12)	−0.0006 (9)	0.0057 (10)
C22	0.0687 (13)	0.0805 (17)	0.0337 (9)	−0.0184 (12)	0.0001 (9)	−0.0061 (10)
C23	0.0566 (11)	0.0521 (13)	0.0364 (9)	0.0007 (9)	0.0014 (8)	−0.0043 (8)
C24	0.0591 (11)	0.0432 (11)	0.0350 (9)	−0.0041 (9)	−0.0003 (8)	−0.0056 (8)
C25	0.0622 (12)	0.0570 (14)	0.0367 (9)	0.0019 (10)	−0.0001 (8)	−0.0033 (9)
C26	0.0814 (15)	0.0658 (16)	0.0414 (10)	−0.0096 (12)	0.0148 (10)	−0.0126 (10)
C27	0.0672 (13)	0.0673 (16)	0.0612 (13)	0.0085 (11)	0.0102 (11)	−0.0137 (11)
C28	0.0638 (12)	0.0570 (14)	0.0471 (11)	0.0064 (10)	−0.0026 (9)	−0.0032 (9)

### Geometric parameters (Å, °)

**Table:**

O1—C1	1.316 (2)	C8—H8A	0.9700
O2—C1	1.194 (3)	C8—H8B	0.9700
O1—H1	0.8200	C9—H9	0.9300
O3—C15	1.307 (2)	C11—H11	0.9300
O4—C15	1.180 (3)	C12—H12	0.9300
O3—H3	0.8200	C13—H13	0.9300
N1—C8	1.459 (2)	C14—H14	0.9300
N1—C9	1.257 (2)	C15—C16	1.510 (3)
N2—C12	1.330 (3)	C16—C17	1.523 (3)
N2—C11	1.340 (2)	C16—C21	1.506 (3)
N3—C22	1.454 (2)	C17—C18	1.534 (3)
N3—C23	1.255 (2)	C18—C19	1.506 (3)
N4—C26	1.328 (3)	C19—C20	1.511 (3)
N4—C25	1.332 (2)	C19—C22	1.531 (3)
C1—C2	1.514 (3)	C20—C21	1.527 (3)
C2—C3	1.529 (3)	C23—C24	1.474 (2)
C2—C7	1.518 (3)	C24—C25	1.390 (3)
C3—C4	1.528 (3)	C24—C28	1.385 (3)
C4—C5	1.517 (3)	C26—C27	1.385 (3)
C5—C6	1.524 (2)	C27—C28	1.366 (3)
C5—C8	1.521 (2)	C16—H16	0.9800
C6—C7	1.525 (3)	C17—H17A	0.9700
C9—C10	1.470 (2)	C17—H17B	0.9700
C10—C11	1.385 (3)	C18—H18A	0.9700
C10—C14	1.391 (3)	C18—H18B	0.9700
C12—C13	1.377 (3)	C19—H19	0.9800
C13—C14	1.374 (3)	C20—H20A	0.9700
C2—H2	0.9800	C20—H20B	0.9700
C3—H3A	0.9700	C21—H21A	0.9700
C3—H3B	0.9700	C21—H21B	0.9700
C4—H4A	0.9700	C22—H22A	0.9700
C4—H4B	0.9700	C22—H22B	0.9700
C5—H5	0.9800	C23—H23	0.9300
C6—H6A	0.9700	C25—H25	0.9300
C6—H6B	0.9700	C26—H26	0.9300
C7—H7B	0.9700	C27—H27	0.9300
C7—H7A	0.9700	C28—H28	0.9300
			
C1—O1—H1	109.00	C14—C13—H13	121.00
C15—O3—H3	109.00	C10—C14—H14	120.00
C8—N1—C9	118.10 (18)	C13—C14—H14	120.00
C11—N2—C12	117.77 (17)	O3—C15—C16	113.09 (17)
C22—N3—C23	118.38 (17)	O4—C15—C16	125.25 (17)
C25—N4—C26	117.31 (18)	O3—C15—O4	121.64 (18)
O1—C1—C2	112.17 (18)	C15—C16—C17	112.64 (17)
O1—C1—O2	122.44 (18)	C17—C16—C21	110.89 (16)
O2—C1—C2	125.39 (18)	C15—C16—C21	111.62 (16)
C3—C2—C7	110.11 (17)	C16—C17—C18	110.72 (18)
C1—C2—C3	112.50 (16)	C17—C18—C19	111.56 (17)
C1—C2—C7	110.97 (16)	C18—C19—C22	111.83 (17)
C2—C3—C4	111.33 (17)	C20—C19—C22	110.96 (19)
C3—C4—C5	113.16 (15)	C18—C19—C20	110.42 (19)
C4—C5—C8	112.17 (15)	C19—C20—C21	112.3 (2)
C4—C5—C6	110.54 (16)	C16—C21—C20	111.28 (19)
C6—C5—C8	110.27 (15)	N3—C22—C19	112.70 (17)
C5—C6—C7	111.90 (15)	N3—C23—C24	121.85 (18)
C2—C7—C6	110.87 (16)	C23—C24—C28	122.30 (17)
N1—C8—C5	112.45 (15)	C25—C24—C28	117.35 (17)
N1—C9—C10	122.50 (19)	C23—C24—C25	120.31 (18)
C11—C10—C14	117.84 (15)	N4—C25—C24	123.8 (2)
C9—C10—C14	122.51 (16)	N4—C26—C27	123.3 (2)
C9—C10—C11	119.64 (18)	C26—C27—C28	118.6 (2)
N2—C11—C10	123.18 (19)	C24—C28—C27	119.66 (18)
N2—C12—C13	123.16 (18)	C15—C16—H16	107.00
C12—C13—C14	118.89 (19)	C17—C16—H16	107.00
C10—C14—C13	119.16 (17)	C21—C16—H16	107.00
C1—C2—H2	108.00	C16—C17—H17A	110.00
C3—C2—H2	108.00	C16—C17—H17B	109.00
C7—C2—H2	108.00	C18—C17—H17A	109.00
C4—C3—H3A	109.00	C18—C17—H17B	110.00
C4—C3—H3B	109.00	H17A—C17—H17B	108.00
C2—C3—H3B	109.00	C17—C18—H18A	109.00
C2—C3—H3A	109.00	C17—C18—H18B	109.00
H3A—C3—H3B	108.00	C19—C18—H18A	109.00
C3—C4—H4A	109.00	C19—C18—H18B	109.00
C3—C4—H4B	109.00	H18A—C18—H18B	108.00
C5—C4—H4B	109.00	C18—C19—H19	108.00
C5—C4—H4A	109.00	C20—C19—H19	108.00
H4A—C4—H4B	108.00	C22—C19—H19	108.00
C8—C5—H5	108.00	C19—C20—H20A	109.00
C6—C5—H5	108.00	C19—C20—H20B	109.00
C4—C5—H5	108.00	C21—C20—H20A	109.00
C7—C6—H6A	109.00	C21—C20—H20B	109.00
C5—C6—H6A	109.00	H20A—C20—H20B	108.00
H6A—C6—H6B	108.00	C16—C21—H21A	109.00
C7—C6—H6B	109.00	C16—C21—H21B	109.00
C5—C6—H6B	109.00	C20—C21—H21A	109.00
C2—C7—H7A	109.00	C20—C21—H21B	109.00
C2—C7—H7B	109.00	H21A—C21—H21B	108.00
C6—C7—H7A	109.00	N3—C22—H22A	109.00
C6—C7—H7B	109.00	N3—C22—H22B	109.00
H7A—C7—H7B	108.00	C19—C22—H22A	109.00
C5—C8—H8B	109.00	C19—C22—H22B	109.00
H8A—C8—H8B	108.00	H22A—C22—H22B	108.00
C5—C8—H8A	109.00	N3—C23—H23	119.00
N1—C8—H8B	109.00	C24—C23—H23	119.00
N1—C8—H8A	109.00	N4—C25—H25	118.00
N1—C9—H9	119.00	C24—C25—H25	118.00
C10—C9—H9	119.00	N4—C26—H26	118.00
N2—C11—H11	118.00	C27—C26—H26	118.00
C10—C11—H11	118.00	C26—C27—H27	121.00
N2—C12—H12	118.00	C28—C27—H27	121.00
C13—C12—H12	118.00	C24—C28—H28	120.00
C12—C13—H13	121.00	C27—C28—H28	120.00
			
C9—N1—C8—C5	−129.8 (2)	C11—C10—C14—C13	0.5 (3)
C8—N1—C9—C10	−177.13 (18)	C9—C10—C14—C13	179.63 (19)
C12—N2—C11—C10	−0.7 (3)	N2—C12—C13—C14	0.3 (3)
C11—N2—C12—C13	0.4 (3)	C12—C13—C14—C10	−0.7 (3)
C22—N3—C23—C24	−173.32 (19)	O3—C15—C16—C17	177.02 (19)
C23—N3—C22—C19	−144.5 (2)	O3—C15—C16—C21	−57.5 (2)
C26—N4—C25—C24	1.0 (3)	O4—C15—C16—C17	−1.4 (3)
C25—N4—C26—C27	−0.5 (3)	O4—C15—C16—C21	124.1 (3)
O1—C1—C2—C3	177.38 (19)	C15—C16—C17—C18	−178.30 (18)
O1—C1—C2—C7	−58.8 (2)	C21—C16—C17—C18	55.8 (2)
O2—C1—C2—C3	−2.5 (3)	C15—C16—C21—C20	178.56 (17)
O2—C1—C2—C7	121.4 (2)	C17—C16—C21—C20	−55.0 (2)
C1—C2—C3—C4	179.77 (18)	C16—C17—C18—C19	−56.6 (2)
C1—C2—C7—C6	177.12 (16)	C17—C18—C19—C20	55.7 (2)
C3—C2—C7—C6	−57.7 (2)	C17—C18—C19—C22	179.75 (17)
C7—C2—C3—C4	55.4 (2)	C18—C19—C20—C21	−55.0 (3)
C2—C3—C4—C5	−53.6 (2)	C22—C19—C20—C21	−179.5 (2)
C3—C4—C5—C6	52.2 (2)	C18—C19—C22—N3	79.5 (2)
C3—C4—C5—C8	175.74 (17)	C20—C19—C22—N3	−156.8 (2)
C4—C5—C8—N1	66.0 (2)	C19—C20—C21—C16	55.1 (3)
C4—C5—C6—C7	−54.0 (2)	N3—C23—C24—C25	−173.0 (2)
C6—C5—C8—N1	−170.34 (18)	N3—C23—C24—C28	9.5 (3)
C8—C5—C6—C7	−178.61 (18)	C23—C24—C25—N4	−178.5 (2)
C5—C6—C7—C2	57.7 (2)	C28—C24—C25—N4	−0.9 (3)
N1—C9—C10—C11	174.7 (2)	C23—C24—C28—C27	177.8 (2)
N1—C9—C10—C14	−4.5 (3)	C25—C24—C28—C27	0.3 (3)
C9—C10—C11—N2	−178.96 (19)	N4—C26—C27—C28	0.0 (4)
C14—C10—C11—N2	0.2 (3)	C26—C27—C28—C24	0.2 (3)

### Hydrogen-bond geometry (Å, °)

**Table:**

*D*—H···*A*	*D*—H	H···*A*	*D*···*A*	*D*—H···*A*
O1—H1···N4^i^	0.82	1.87	2.682 (2)	171
O3—H3···N2^ii^	0.82	1.89	2.685 (2)	164
C11—H11···O2^iii^	0.93	2.56	3.478 (3)	168
C13—H13···O2^iv^	0.93	2.57	3.316 (3)	137
C27—H27···O4^v^	0.93	2.45	3.280 (3)	148

Symmetry codes: (i) *x*, *y*, *z*+1; (ii) *x*+1/2, −*y*+1/2, *z*+1/2; (iii) *x*−1/2, −*y*+1/2, *z*−1/2; (iv) −*x*+1, −*y*, −*z*+1; (v) −*x*+3/2, *y*−1/2, −*z*+1/2.
